# Interaction of Norepinephrine and Glucocorticoids Modulate Inhibition of Principle Cells of Layer II Medial Entorhinal Cortex in Male Mice

**DOI:** 10.3389/fnsyn.2018.00003

**Published:** 2018-03-28

**Authors:** Jeremiah P. Hartner, Laura A. Schrader

**Affiliations:** ^1^Neuroscience Program, Tulane Brain Institute, Tulane University, New Orleans, LA, United States; ^2^Department of Cell and Molecular Biology, Tulane University, New Orleans, LA, United States

**Keywords:** inhibitory interneurons, grid cells, stellate cells, pyramidal cells, slice preparation, stress, psychological, memory

## Abstract

Spatial memory processing requires functional interaction between the hippocampus and the medial entorhinal cortex (MEC). The grid cells of the MEC are most abundant in layer II and rely on a complex network of local inhibitory interneurons to generate spatial firing properties. Stress can cause spatial memory deficits in males, but the specific underlying mechanisms affecting the known memory pathways remain unclear. Stress activates both the autonomic nervous system and the hypothalamic-pituitary-adrenal axis to release norepinephrine (NE) and glucocorticoids, respectively. Given that adrenergic receptor (AR) and glucocorticoid receptor (GR) expression is abundant in the MEC, both glucocorticoids and NE released in response to stress may have rapid effects on MEC-LII networks. We used whole-cell patch clamp electrophysiology in MEC slice preparations from male mice to test the effects of NE and glucocorticoids on inhibitory synaptic inputs of MEC-LII principal cells. Application of NE (100 μM) increased the frequency and amplitude of spontaneous inhibitory post-synaptic currents (sIPSCs) in approximately 75% of the principal cells tested. Unlike NE, bath application of dexamethasone (Dex, 1 μM), a synthetic glucocorticoid, or corticosterone (1 μM) the glucocorticoid in rodents, rapidly decreased the frequency of sIPSCs, but not miniature (mIPSCs) in MEC-LII principal cells. Interestingly, pre-treatment with Dex prior to NE application led to an NE-induced increase in sIPSC frequency in all cells tested. This effect was mediated by the α1-AR, as application of an α1-AR agonist, phenylephrine (PHE) yielded the same results, suggesting that a subset of cells in MEC-LII are unresponsive to α1-AR activation without prior activation of GR. We conclude that activation of GRs primes a subset of principal cells that were previously insensitive to NE to become responsive to α1-AR activation in a transcription-independent manner. These findings demonstrate the ability of stress hormones to markedly alter inhibitory signaling within MEC-LII circuits and suggest the intriguing possibility of modulation of network processing upstream of the hippocampus.

## Introduction

Stress and stress hormones impair spatial memory in male animals, and the function of the hippocampus is known to be affected by stress hormones. The entorhinal cortex is highly interconnected with the hippocampal formation, and interactions between the hippocampus and medial entorhinal cortex (MEC) are important for spatial memory and consolidation (Sanders et al., 2015). Specifically, MEC receives inputs from visual/spatial areas of the brain (Burwell and Amaral, 1998a,b; Kerr et al., 2007; Agster and Burwell, 2013), and most cells of the MEC are spatially selective (Fyhn et al., 2004; Diehl et al., 2017). The grid cells of the MEC are important for encoding spatial cognition in normal conditions (Hafting et al., 2005; Gil et al., 2018), and layer II of the MEC (MEC-LII) contains a high density of grid cells that project to the hippocampus (Hafting et al., 2005). Dysfunction of MEC is also implicated in several neuropathological conditions, including epilepsy (de Curtis and Pare, 2004), schizophrenia (Berretta et al., 2015), and Alzheimer’s disease, as the layer II of the entorhinal cortex is one of the first areas to show neurodegenerative effects of Alzheimer’s disease (Alonso and Klink, 1993; Schousboe et al., 1993). Therefore, understanding modulation of this circuitry is crucial to understanding both normal pathological brain function.

Medial entorhinal cortex – LII contains 2 general classes of principal cells: stellate and pyramidal cells (Fuchs et al., 2016). Stellate cells send projections to the dentate gyrus (DG) of the hippocampus, whereas pyramidal cells project directly to CA1 and the contralateral MEC (Varga et al., 2010). MEC-LII also contains multiple inhibitory cell classes including parvalbumin (PV)^+^ fast spiking interneurons (FSIs), cholecystokinin basket cells CCKBCs, and somatostatin (SOM)^+^ interneurons (Miettinen et al., 1996; Lee et al., 2010; Varga et al., 2010). The connectivity between the principal cells and inhibitory interneurons forms a dense and complex network, but the mechanisms of modulation of these networks, and specifically the effects of stress hormones, are not well understood.

An organism’s response to stress involves activation of two systems: the hypothalamo-pituitary-adrenal (HPA) axis and the sympathetic nervous system. Activation of the HPA axis causes release of glucocorticoids into the general circulation, which readily cross the blood–brain barrier and can act on both glucocorticoid receptors (GRs) and mineralocorticoid receptors (MRs). The medial entorhinal cortex (MEC) contains GRs (Sarrieau et al., 1988); however, the effect of GR activation on signaling in the MEC remains unstudied. In addition to activation of the HPA axis, stress also activates the autonomic nervous system to release NE from the neurons of the locus coeruleus and brainstem solitary tract nucleus (NTS) (Loizou, 1969; Jones et al., 1977; Fallon et al., 1978; Palkovits et al., 1979; Gold, 2015; Herman, 2017). Noradrenergic efferents primarily from the locus coeruleus (LC) regulate neuronal function in a variety of areas including those that are crucial for learning and memory (Gibbs and Summers, 2002; Roozendaal and McGaugh, 2011).

The entorhinal cortex expresses α1 (Stanton et al., 1987), α2 (Unnerstall et al., 1984; Boyajian et al., 1987), and β-adrenergic receptors (ARs) (Booze et al., 1993), and inhibitory interneurons throughout the brain express both α1 and β-ARs (Papay et al., 2006; Cox et al., 2008). In prepubertal male rodents, NE activates α1-ARs and significantly increases sIPSC frequency and amplitude as well as mIPSC frequency, but not mIPSC amplitude in principal cells in MEC-LII and LIII (Lei et al., 2007). NE application also reduces action potential firing in more than 50% of MEC-LII and LIII principal cells (Lei et al., 2007; Xiao et al., 2009). Taken together, the effects of AR activation in the MEC may demonstrate a crucial role of the connection between sympathetic nervous system activation and spatial memory processing deficits.

Given that both stellate and pyramidal cell classes are connected directly to hippocampal subregions (Varga et al., 2010) and possess intrinsic properties demonstrating the ability to encode spatial information (Alonso and Klink, 1993; Hafting et al., 2005; Domnisoru et al., 2013; Tang et al., 2014), spatial memory processing could be affected by both stellate and pyramidal cell signaling. Because stellate cells are connected to each other exclusively through inhibitory interneurons and do not appear to form excitatory connections with other LII principal cells (Couey et al., 2013; Pastoll et al., 2013), along with the fact that MEC-LII has an extensive and relatively strong inhibitory network interwoven with principal cells, alteration to signaling between principal cells and local inhibitory interneurons is the most likely mechanism for spatial processing disruption underlying the link between stress and spatial memory deficits.

In the present study, we tested the effect of both glucocorticoids and NE on inhibitory signaling in MEC – LII principal cells. Our results demonstrate that NE applied alone can significantly increase the frequency and amplitude of spike-driven IPSCs as well as frequency of terminal-specific mIPSCs in MEC – LII principal cells, and these effects are primarily mediated by α1-AR activation. Glucocorticoid application alone rapidly reduced the frequency of spike-dependent spontaneous inhibitory post-synaptic currents (sIPSCs). Interestingly, co-administration of glucocorticoids and NE produces a synergistic effect in MEC-LII principal cells that is unlikely to be due to genomic changes, whereby a population of cells previously insensitive to NE is primed to be NE-sensitive after 15 min of Dex application.

## Materials and Methods

The Tulane University Institutional Animal Care and Use Committee (IACUC) approved all procedures. C57Bl/6 male mice were obtained from Charles River. All animals were group-housed on a normal light dark cycle (lights on -7 am-7 pm) in a standard enriched environment and fed *ad libitum*. Slices were made from mice 4–8 weeks old (>90% were ∼5 weeks). After at least 1 week of habituation, mice were anesthetized with isoflurane (VetOne) inhalation and decapitated. The mouse brains were immersed in 0–1°C NMDG-containing artificial cerebrospinal fluid (ACSF) cutting solution composed of (in mM): 110 NMDG, 110 HCl, 3 KCl, 10 MgCl_2_, 1.1 NaH_2_P0_4_, 0.5 CaCl_2_, 25 glucose, 3 pyruvic acid, 10 ascorbic acid, and 25 NaHCO_3_, with an osmolarity of 305–315 mOsm/L and a pH of 7.2–7.3. Para-sagittal slice preparations (Pastoll et al., 2012) of 300 μm thickness were prepared and transferred to a storage chamber where they were maintained at room temperature in carboxygen-bubbled physiological ACSF containing (in mM): 124 NaCl, 2.5 KCl, 25 NaHCO_3_, 1.2 NaH_2_PO_4_, 20 Glucose, 1 MgCl_2_, 2 CaCl_2_, with an osmolarity of 290–300 mOsm/L and a pH of 7.2–7.3.

### Electrophysiological Recordings

Medial entorhinal cortex slices were transferred to submersion recording chamber continuously perfused with 34–37°C ACSF. Whole-cell patch clamp recordings of principal cells were achieved in dorsal MEC – LII using a MultiClamp 700B amplifier (Molecular Devices) at a holding potential of -65 mV. Patch pipettes were formed on a horizontal puller (P97; Sutter Instruments) with a tip resistance of 2–6 MΩ. To target layer II principal cells, all patched cells were large, polygonal or triangular in shape (Canto et al., 2008) and located at the dorsal most portion of MEC-LII and located near (generally within one cell body width) the superficial edge of MEC-LII in order to exclude patching MEC-LIII cells.

For the majority of inhibitory post-synaptic current (IPSC) recordings, patch electrodes were filled with a high chloride intracellular solution containing (in mM): 120 CsCl, 30 HEPES, 2 MgCl_2_, 1 CaCl_2_, 11 EGTA, 4 ATP-Mg, with an osmolarity of 300–310 mOsm/L and a pH adjusted to 7.2–7.3 with CsOH. APV (50 μM) and DNQX (20 μM) were added to the bath to block excitatory glutamate receptor-mediated transmission. Tetrodotoxin (TTX, 1 μM) was added to the bath when recording miniature IPSCs to block spike-evoked glutamate release. Because CsCl is known to block potassium channels and does not allow for accurate recording of intrinsic cellular properties including membrane potential and input resistance, some IPSC recordings were performed with patch electrodes containing a high chloride intracellular solution without CsCl (in mM): 135 KCl, 10 HEPES, 2 Na-ATP, 0.2 Na-GTP, 2 MgCl_2_, and 0.1 EGTA, with an osmolarity of 300–310 mOsm/L and a pH adjusted to 7.2–7.4 with KOH.

Following achievement of whole-cell access, the cell stabilized for 5 min prior to recording. Only cells with an access resistance of less than 20 MΩ and less than 20% change in access resistance over the course of the recording were used. Recording conditions for AR activation were performed with bath perfusion of (NE, 100 μM), phenylephrine (PHE, 100 μM), UK14304 (1 μM), isoprenaline (1 μM), or prazosin (10 μM). IPSCs were recorded in voltage clamp at a holding potential of -65 mV for a minimum of 5 min in control conditions prior to infusion of ACSF containing NE or any of the AR agonists. Experimental conditions were achieved for a minimum of 10 min to ensure maximal drug effect. Glucocorticoid experimental recording conditions were performed with either Dex (10 nM – 1 μM) or corticosterone (1 μM) perfused into the bath. IPSCs were recorded in voltage clamp at a holding potential of -65 mV for a minimum of 2 min in control condition prior to infusion of ACSF containing Dex or corticosterone (Cort). Experimental conditions were achieved for a minimum of 10 min to ensure maximal drug effect.

During testing for differential effects of co-administration of glucocorticoids and NE, experimental recording conditions were performed with NE (100 μM), Dex (1 μM), or both, perfused into the bath. IPSCs were recorded in voltage-clamp at a holding potential of -65 mV for a minimum of 5 min in control conditions prior to infusion of ACSF containing either NE or Dex. Experimental conditions were achieved for a minimum of 10 min to ensure maximal drug effect prior to adding the second drug, resulting in both NE and Dex being perfused into the bath together following 10 min of the first experimental condition.

Recordings of synaptic activity were analyzed using MiniAnalysis. Comparisons of 1-min averages representative of the control and maximum drug effect synaptic activity frequency, inter-event interval, amplitude, and decay time between control and drug-treated cells were calculated using paired *t*-test between control and drug conditions. The effects of drugs combined (i.e., control, Dex, and Dex+NE) were calculated using a one-way analysis of variance (ANOVA) test followed by Tukey’s multiple comparisons test. Comparisons between Dex priming of the NE response vs. Dex priming of the phenylephrine response were calculated using a two-way ANOVA. All statistical tests were performed in GraphPad Prism. *P*-values < 0.05 were considered significant.

## Results

### Norepinephrine Increased Frequency and Amplitude of Inhibitory Signaling in a Majority of MEC – LII Principal Cells

**FIGURE 1:**
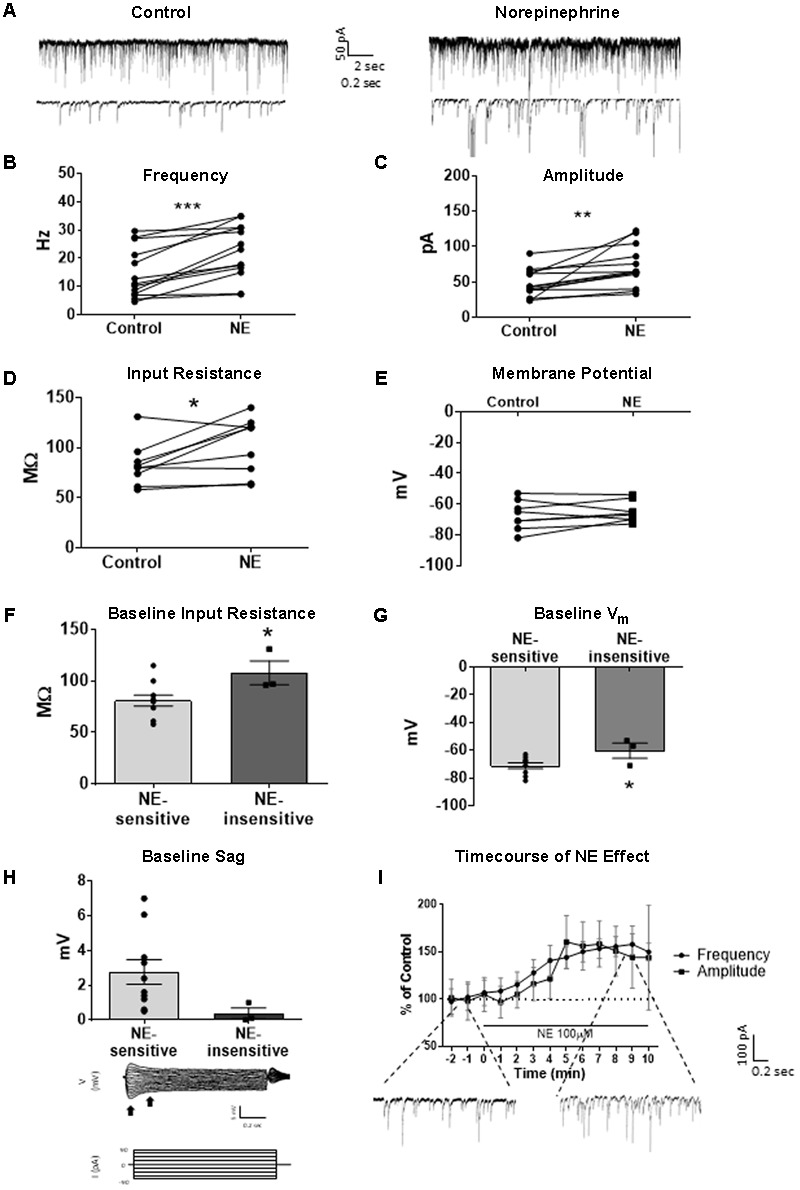
Norepinephrine (100 μM) increases spike-dependent IPSC frequency, amplitude, and input resistance in a subset of principal neurons. **(A)** 20 s (top) and 2 s (bottom) of sIPSC voltage-clamp recordings with KCl intracellular solution representative of control (left) and NE (right) conditions (*n* = 13). **(B)** NE significantly increased average sIPSC frequency. **(C)** NE significantly increased sIPSC amplitude. **(D)** NE significantly increased average input resistance but had no effect on membrane potential (*n* = 9) **(E). (F)** Comparison of baseline input resistance in cells that show >15% increase in sIPSC frequency (*n* = 10) vs. cells that show no change (*n* = 3) in sIPSC frequency. **(G)** Comparison of baseline membrane potential in cells that show >15% increase in sIPSC frequency (*n* = 10) vs. cells that show no change (*n* = 3). Note that the NE-insensitive group has a significantly depolarized average baseline membrane potential in comparison to the NE-sensitive group. **(H)** Comparison of baseline sag amplitude in cells that show >15% increase in sIPSC frequency (*n* = 10) vs. cells that show no change (*n* = 3). Note that the NE-sensitive group has larger average baseline sag, though the difference is not significant potentially due to the low number of cells in the NE-insensitive group. Below: Example trace showing sag response (peak vs. steady-state indicated by black arrows) due to *I*_h_ activation following hyperpolarizing steps in voltage clamp. **(I)** Time course of the effect of NE on frequency and amplitude of sIPSCs in 1-min intervals. NE enters the bath at time 0. Insets show representative voltage clamp recordings with CsCl intracellular solution. Data values shown are the average of each group ± SEM control was compared to NE using paired *t*-test. NE sensitive and insensitive cells were compared using unpaired *t*-test (^∗^*p* < 0.05, ^∗∗^*p* < 0.01, ^∗∗∗^*p* < 0.001).

We tested the effects of NE in the principal cells of layer II of the MEC. Using a KCl-based internal solution, control sIPSCs were recorded at -65 mV in MEC-LII principal cells (**Figure 1A**). Ten minutes NE (100 μM) application significantly increased sIPSC frequency (*p* = 0.0005; **Table 1**) and amplitude (*p* = 0.008; **Table 2**), but not decay time (*p* = 0.06; **Table 3**) (**Figures 1B,C**). Importantly, 3 of the 13 (∼23%) cells showed no change (less than 15% change from control) in sIPSC frequency following NE application (**Tables 1–3**). These cells will be referred to as NE-insensitive cells in the following sections.

**Table 1:** Effect of adrenergic receptor activation on IPSC frequency.

Recording condition	Control frequency (Hz)	NE/agonist frequency (Hz)	*t*	df	*p*	% of control	*t*	df	*p*
sIPSCs NE (KCl int.)	14.65 ± 2.49	22.32 ± 2.65	4.77	12	0.0005^∗^	177.19 ± 22.21	3.48	12	0.005^∗^
sIPSCs NE	42.39 ± 3.89	54.32 ± 3.59	5.15	12	0.0002^∗^	134.83 ± 8.31	4.19	12	0.001^∗^
mIPSCs NE	15.09 ± 1.56	20.73 ± 2.35	4.85	18	0.0001^∗^	139.65 ± 6.19	6.41	18	<0.0001^∗^
sIPSCs PHE	29.93 ± 4.33	36.39 ± 5.70	1.89	9	0.09	121.57 ± 13.84	1.56	9	0.15
mIPSCs PHE	8.19 ± 2.29	8.11 ± 1.65	0.1	5	0.93	111.02 ± 8.98	1.23	5	0.27
sIPSCs UK14304	35.25 ± 7.46	32.12 ± 6.20	1.78	6	0.13	93.54 ± 3.95	1.63	6	0.15
mIPSCs UK14304	14.12 ± 1.45	14.58 ± 1.27	0.34	5	0.75	106.05 ± 7.05	0.69	5	0.52
sIPSCs Isoprenaline	19.12 ± 4.14	18.62 ± 3.49	0.45	4	0.67	101.75 ± 3.83	0.36	4	0.74
mIPSCs Isoprenaline	12.97 ± 3.63	13.19 ± 2.98	0.19	6	0.86	105.94 ± 6.78	0.88	6	0.40

*^∗^*p* < 0.05.*

**Table 2:** Effect of adrenergic receptor activation on IPSC amplitude.

Recording condition	Control amplitude (pA)	NE/agonist amplitude (pA)	*t*	df	*p*	% of control	*t*	df	*p*
sIPSCs NE (KCl int.)	48.04 ± 5.54	71.90 ± 8.12	3.2	12	0.008^∗^	167.04 ± 30.27	2.22	12	0.047^∗^
sIPSCs NE	57.90 ± 9.34	80.61 ± 11.57	4.61	12	0.0006^∗^	148.99 ± 12.66	3.87	12	0.002^∗^
mIPSCs NE	41.35 ± 1.82	42.20 ± 2.06	1.24	18	0.23	101.94 ± 1.77	1.1	18	0.29
sIPSCs PHE	57.09 ± 4.02	60.66 ± 9.19	0.48	9	0.64	105.49 ± 11.68	0.47	9	0.65
mIPSCs PHE	31.90 ± 2.52	30.28 ± 3.75	1.1	5	0.32	93.32 ± 5.65	1.18	5	0.29
sIPSCs UK14304	59.81 ± 8.84	53.55 ± 3.98	0.78	6	0.47	97.62 ± 9.87	0.24	6	0.82
mIPSCs UK14304	39.81 ± 1.75	38.91 ± 2.27	0.7	5	0.52	97.59 ± 2.56	0.81	5	0.45
sIPSCs Isoprenaline	59.74 ± 6.50	59.52 ± 10.39	0.03	4	0.98	97.77 ± 9.00	0.2	4	0.85
mIPSCs Isoprenaline	35.78 ± 1.18	35.92 ± 1.25	0.12	6	0.91	100.63 ± 3.17	0.20	6	0.85

*^∗^*p* < 0.05.*

**Table 3:** Effect of adrenergic receptor activation on IPSC decay time.

Recording condition	Control decay time (ms)	NE/agonist decay time (ms)	*t*	df	*p*	% of control	*t*	df	*p*
sIPSCs NE (KCl int.)	7.13 ± 0.48	7.46 ± 0.54	2.11	12	0.06	104.47 ± 2.27	1.97	12	0.07
sIPSCs NE	8.91 ± 0.50	9.18 ± 0.52	0.73	12	0.48	103.93 ± 3.92	1	12	0.34
mIPSCs NE	7.08 ± 0.25	7.80 ± 0.29	6.03	18	<0.0001^∗^	110.44 ± 1.57	6.67	18	<0.0001^∗^
sIPSCs PHE	8.62 ± 0.50	9.16 ± 0.49	1.79	9	0.11	107.04 ± 3.50	2.01	9	0.08
mIPSCs PHE	8.08 ± 1.07	8.64 ± 0.97	1.9	5	0.16	108.55 ± 4.40	1.95	5	0.11
sIPSCs UK14304	8.28 ± 0.43	8.06 ± 0.28	0.96	6	0.37	98.03 ± 2.81	0.7	6	0.51
mIPSCs UK14304	6.76 ± 0.46	7.32 ± 0.46	4.92	5	0.004^∗^	108.52 ± 1.63	4.53	5	0.006^∗^
sIPSCs Isoprenaline	7.46 ± 0.65	7.92 ± 0.66	1.33	4	0.25	106.57 ± 3.72	1.4	4	0.24
mIPSCs Isoprenaline	7.79 ± 0.35	8.12 ± 0.27	2.01	6	0.09	104.68 ± 2.35	1.99	6	0.07

*^∗^*p* < 0.05.*

Use of KCl-based high-chloride internal solution allowed for recording of intrinsic cellular characteristics in control and NE conditions. NE significantly increased the average input resistance compared to control conditions (*p* = 0.03) (**Figure 1D**), but NE did not affect the average membrane potential (*p* = 0.39) in MEC-LII principal cells (**Figure 1E**). Interestingly, NE-insensitive cells (<+15% change in IPSC frequency following NE application) had a significantly larger average baseline input resistance when compared to NE-sensitive cells (*p* = 0.04) (**Figure 1F**) and the NE-insensitive group had a significantly depolarized average baseline membrane potential in comparison to the NE-sensitive group (*p* = 0.04) (**Figure 1G**). Average baseline sag amplitude in MEC-LII principal cells was larger in cells with an NE-induced increase in sIPSC frequency than NE-insensitive cells, but the difference was not significant (*p* = 0.10) (**Figure 1H**).

A CsCl-based internal solution was used for the remainder of the experiments. We first confirmed that the above effect of NE on MEC-LII principal cell sIPSCs was conserved when recording with CsCl-based internal solution. Spontaneous IPSCs (sIPSCs) were recorded at a holding potential of -65 mV in a CsCl-based high-chloride internal solution. NE significantly increased sIPSC frequency (*p* = 0.0002; **Table 1**) and sIPSC amplitude (*p* = 0.0006; **Table 2**), but not decay time (*p* = 0.48; **Table 3**). NE increased average sIPSC frequency and amplitude within the 1st minute of perfusion, and maximum effect on frequency and amplitude occurred within 5–9 min of commencement of NE application (**Figure 1I**). It is important to note that, like the recordings with KCl, 3 of the 13 (∼23%) cells were unaffected (less than15% change from control) in terms of sIPSC frequency following NE application.

**FIGURE 2:**
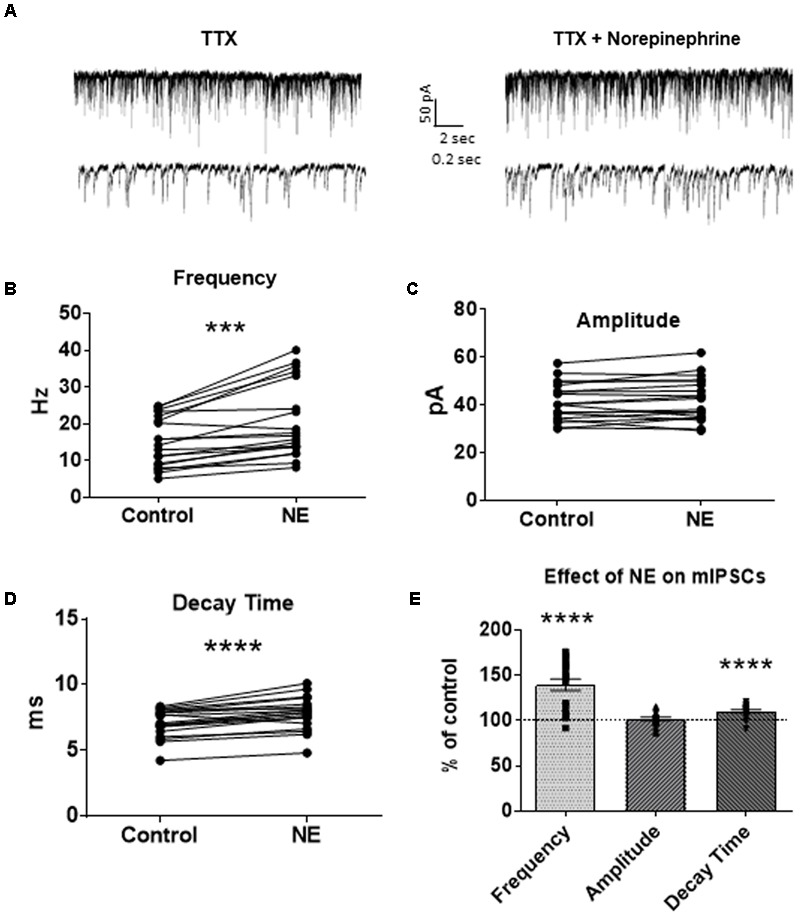
Norepinephrine significantly increased miniature IPSC frequency and decay time, but not amplitude, in MEC – LII principal cells. **(A)** Twenty seconds (Top) and 2 s (Bottom) of mIPSC voltage-clamp recordings in the presence of TTX representative of control (left) and NE (right) conditions. **(B)** NE significantly increased average mIPSC frequency. **(C)** NE did not significantly alter m IPSC amplitude. **(D)** NE significantly increased average mIPSC decay time. **(E)** NE significantly increased mIPSC frequency and decay time, but not amplitude, when normalized and compared to the corresponding control group average (*n* = 19). (^∗∗∗^*p* < 0.001, ^∗∗∗∗^*p* < 0.0001).

To investigate if the NE-induced increase in sIPSC frequency and amplitude is exclusive to spike-driven signaling or also causes changes to terminal-specific inhibitory signaling, miniature IPSCs were recorded from MEC – LII principal cells in the presence of TTX, a voltage-gated sodium channel blocker (**Figure 2A**). NE application significantly increased mIPSC frequency (*p* = 0.0001; **Table 1**) and decay time (*p* < 0.0001; **Table 3**), but failed to significantly alter mIPSC amplitude (*p* = 0.23; **Table 2**) (**Figures 2B–E**). Interestingly, 5 of the 19 cells (∼26%) recorded were unaffected (less than 15% change from control) by NE application (**Tables 1–3**).

### Adrenergic Receptor Subtype That Mediates the NE Effect

**FIGURE 3:**
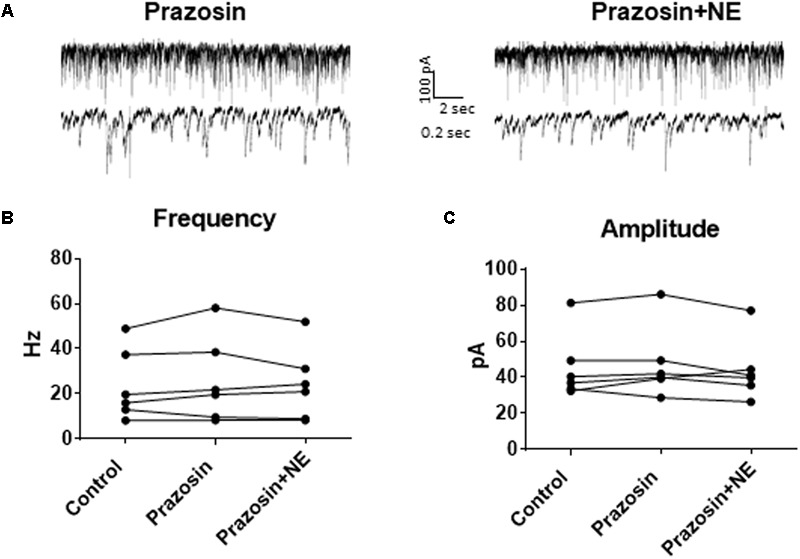
Pre-treatment with prazosin, a specific α1AR antagonist blocks NE-induced sIPSC frequency and amplitude increases. **(A)** Twenty seconds (top) and 2 s (bottom) of sIPSC voltage-clamp recordings in prazosin (left) and prazosin + NE (right) conditions. Prazosin alone had no significant effect on sIPSC frequency **(B)** or amplitude **(C)**, and prazosin blocked the effects of NE on frequency **(B)** and amplitude **(C)** (*n* = 6).

In order to determine the receptor subtypes that mediate the NE effect, prazosin, an α1-AR antagonist (Jurgens et al., 2007), was applied prior to NE. After 10 min of perfusion of the α1-AR antagonist, prazosin (10 μM), sIPSCs were recorded for 15 min (**Figure 3A**). There were no differences between the control, prazosin, and prazosin+NE groups for sIPSC frequency (*p* = 0.46), amplitude (*p* = 0.34), or decay time (*p* = 0.59) (**Figures 3B,C**), indicating that prazosin blocked the NE-induced increase in sIPSC frequency and amplitude. These results suggest that activation of α1-adrenoreceptors is necessary for the NE-induced increase in frequency and amplitude of IPSCs.

**FIGURE 4:**
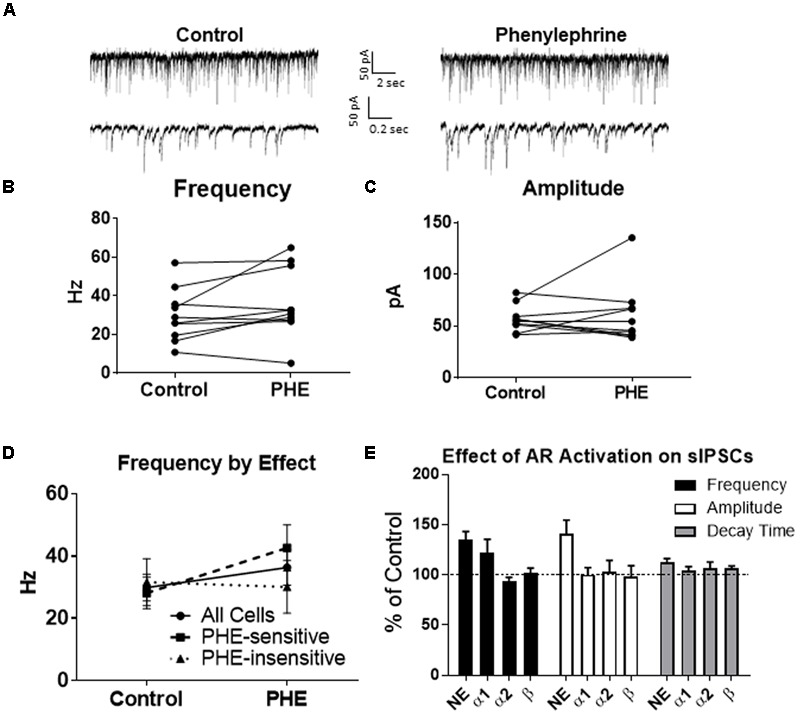
Phenylephrine, an agonist of α1 adrenoreceptors increased spontaneous IPSC frequency in a subset of neurons, and had no effect on amplitude or decay time. **(A)** Twenty seconds (Top) and 2 s (Bottom) of sIPSC voltage-clamp recordings representative of control (left) and PHE (right) conditions. **(B)** PHE failed to significantly increase average sIPSC frequency (*n* = 10). **(C)** PHE had no significant effect on average sIPSC amplitude. **(D)** PHE significantly increased average sIPSC frequency in the 5 PHE-sensitive cells, but had no significant effect in the other five cells. **(E)** summary plots of the average effect of agonists of the adrenoreceptors on spontaneous IPSCs. NE significantly increased sIPSC frequency and amplitude and this was partially mimicked by phenylephrine, the α1AR agonist.

We determined if activation of the α1 receptors could mimic the NE affect. Phenylephrine, an α1-adrenoreceptor agonist (Jurgens et al., 2007; Ferry et al., 2015), was tested (**Figure 4**). Ten minutes of phenylephrine (PHE, 100 μM) perfusion failed to significantly increase sIPSC frequency (*p* = 0.09; **Table 1**) and had no effect on sIPSC amplitude (*p* = 0.64; **Table 2**) or decay time (*p* = 0.11; **Table 3**) (**Figures 4A–C**). It is important to note that PHE caused marked frequency increases (>15%) in 5 of the 10 cells recorded, which measured as significantly higher than control conditions (*p* = 0.03, **Figure 4D**). Five of the 10 cells showed no change in sIPSC frequency following PHE application. These results suggest that in a subset of cells, activation of α1-adrenoreceptors is sufficient to mimic the NE-induced increase in sIPSC frequency in a subset of cells. To determine if PHE also affected terminal-specific inhibitory signaling, mIPSCs were recorded from MEC – LII principal cells in the presence of TTX. PHE had no significant effect on average mIPSC frequency (*p* = 0.93; **Table 1**), amplitude (*p* = 0.32; **Table 2**), or decay time (*p* = 0.16; **Table 3**).

Since phenylephrine did not fully account for the NE effects, we also tested activation of the α2-adrenoreceptor with UK14304 (Jurgens et al., 2007; Ferry et al., 2015). UK14304 had no effect on sIPSC frequency (*p* = 0.13; **Table 1**), amplitude (*p* = 0.47; **Table 2**) or decay time (*p* = 0.37; **Table 3**), indicating that UK14304 has no effect on spike-dependent spontaneous inhibitory signaling in MEC – LII principal cells. To investigate the effect of UK14304 on terminal-specific inhibitory signaling, mIPSCs were recorded from MEC – LII principal cells in the presence of TTX. After 10 min of UK14304 (1 μM) perfusion, mIPSCs were recorded. UK14304 had no effect on average mIPSC frequency (*p* = 0.75; **Table 1**) or amplitude (*p* = 0.52; **Table 2**), but significantly increased average mIPSC decay time (*p* = 0.004; **Table 3**).

We also tested the effects of activation of β-ARs. Isoprenaline 1 μM (Wójtowicz et al., 2010) a β-AR agonist had no significant effect on sIPSC frequency (*p* = 0.67; **Table 1**), amplitude (*p* = 0.98; **Table 2**), or decay time (*p* = 0.25; **Table 3**), indicating that isoprenaline has no effect on spike-dependent inhibitory signaling in MEC – LII principal cells. To investigate the effect of isoprenaline on terminal-specific inhibitory signaling, mIPSCs were recorded from MEC – LII principal cells in the presence of TTX. After 10 min of isoprenaline (1 μM) perfusion, mIPSCs were recorded. Isoprenaline had no effect on average mIPSC frequency (*p* = 0.86; **Table 1**), amplitude (*p* = 0.91; **Table 2**), or decay time (*p* = 0.09; **Table 3**). The overall effect of NE and receptor subtype agonists are shown in **Figure 4E**. Activation of α1-AR agonists mediated an increase in sIPSC frequency that mimicked the effects of NE.

### Glucocorticoids Decrease sIPSC Frequency

**FIGURE 5:**
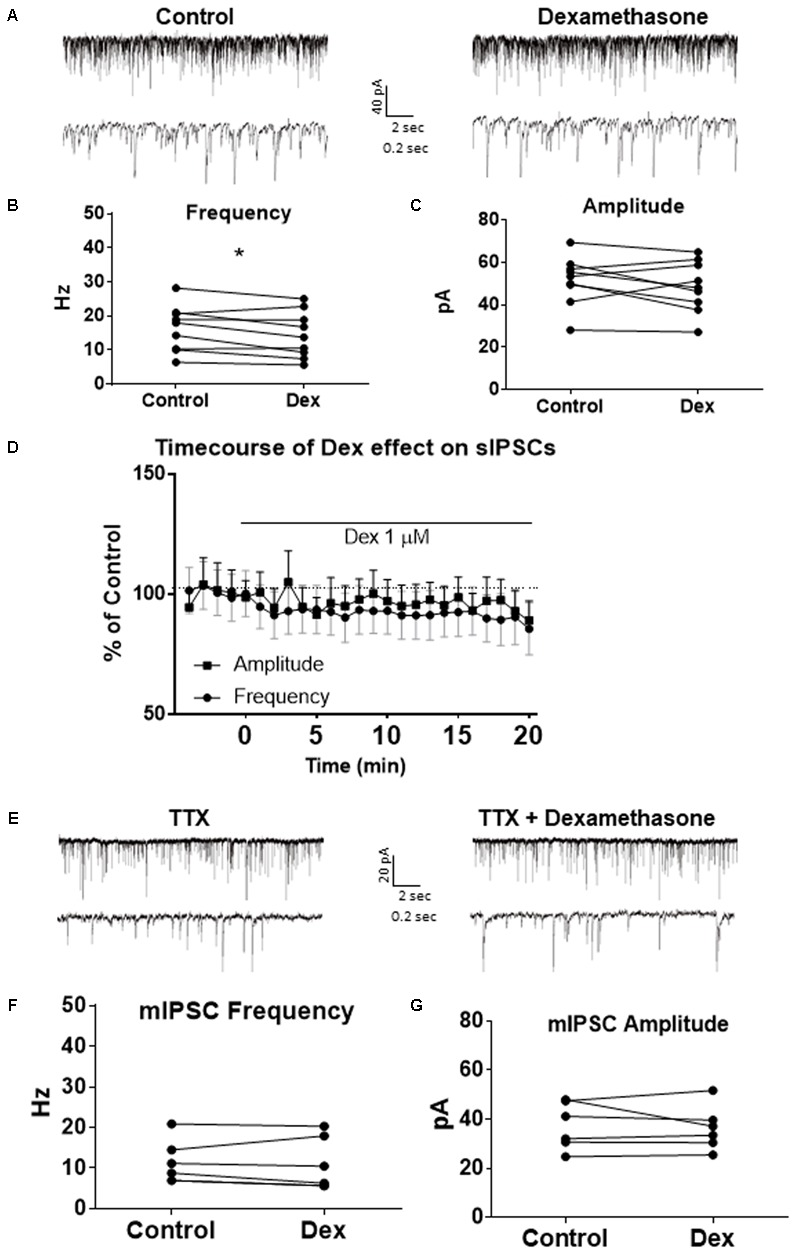
Dexamethasone significantly decreased sIPSC frequency but had no effect on mIPSC frequency. **(A)** Top- 20 s (top) and 2 s (bottom) of sIPSC voltage-clamp recordings representative of control (left) and Dex (right) conditions. **(B)** Dex significantly decreased sIPSC frequency. **(C)** Dex had no effect on sIPSC amplitude. **(D)** Time course of the Dex effect in 1-min intervals. Dex enters at 0 min and quickly decreases frequency, but not amplitude (*n* = 9). **(E)** 20 s (top) and 2 s (bottom) of mIPSC voltage-clamp recordings representative of control (TTX, left) and TTX + Dex (right) conditions. **(F)** Dex had no effect on mIPSC frequency. **(G)** Dex had no effect on mIPSC amplitude (*n* = 6) (^∗^*p* < 0.05).

To test the effects of glucocorticoids on IPSC frequency, we tested the effects of Dex, a synthetic glucocorticoid. Spike-driven spontaneous IPSCs (sIPSCs) were recorded at a holding potential of -65 mV in control and after 10 min Dex application (**Figure 5A**). Dex significantly decreased sIPSC frequency (*p* = 0.04; **Table 4**) but did not significantly alter sIPSC amplitude (*p* = 0.31; **Table 5**) or decay time (*p* = 0.49; **Table 6**) (**Figures 5B,C**). This result suggests that Dex significantly decreased the frequency of spontaneous spike-dependent inhibitory signaling onto MEC – LII principal cells without significantly affecting amplitude or decay time of inhibitory synaptic events.

**Table 4:** Effect of glucocorticoids on frequency of inhibitory synaptic transmission.

Recording type	Control (Hz ± SEM)	Dex -1 μM (Hz ± SEM)	*t*	df (*n*-1)	*p*	% of control	*t*	df	*p*
sIPSCs Dex – 1 μM	16.38 ± 2.26	14.4 ± 2.28	2.42	8	0.04^∗^	87.01 ± 4.94	2.63	8	0.03^∗^
sIPSCs Dex – 10 nM	21.74 ± 4.92	19.21 ± 4.18	0.84	5	0.44	94.82 ± 9.79	0.53	5	0.62
sIPSCs Dex – 100 nM	30.87 ± 4.74	31.60 ± 5.71	0.28	8	0.79	99.88 ± 7.39	0.02	8	0.99
mIPSCs	11.49 ± 2.21	11.01 ± 2.67	0.58	5	0.59	91.51 ± 7.50	1.13	5	0.31
sIPSCs Cort -1 μM	26.55 ± 3.46	22.44 ± 3.90	3.5	7	0.01^∗^	81.05 ± 5.6	3.39	7	0.01^∗^

*^∗^*p* < 0.05.*

**Table 5:** Effect of glucocorticoids on amplitude of inhibitory synaptic transmission.

Recording type	Control (pA ± SEM)	Dex – 1 μM (pA ± SEM)	*t*	df (*n*-1)	*p*	% of control	*t*	df	*p*
sIPSCs Dex – 1 μM	51.37 ± 3.87	48.45 ± 4.04	1.08	8	0.31	95.11 ± 5.41	0.91	8	0.39
sIPSCs Dex – 10 nM	58.34 ± 9.44	60.66 ± 12.27	0.42	5	0.69	103.56 ± 7.85	0.45	5	0.67
sIPSCs Dex – 100 nM	70.83 ± 9.19	62.45 ± 8.28	1.33	8	0.22	90.38 ± 7.17	1.34	8	0.22
mIPSCs	37.31 ± 3.93	36.28 ± 3.69	0.49	5	0.65	98.24 ± 4.53	0.39	5	0.71
sIPSCs Cort – 1 μM	54.85 ± 5.7	47.65 ± 4.72	1.96	7	0.09	89.21 ± 5.86	1.84	7	0.11

**Table 6:** Effect of glucocorticoids on decay time of inhibitory synaptic transmission.

Recording type	Control (ms ± SEM)	Dex – 1 μM (ms ± SEM)	*t*	df (*n*-1)	*p*	% of control	*t*	df	*p*
sIPSCs Dex – 1 μM	7.99 ± 1.0	7.70 ± 0.72	0.73	8	0.49	101.39 ± 6.91	0.2	8	0.85
sIPSCs Dex – 10 nM	7.46 ± 0.62	7.62 ± 0.56	0.86	5	0.43	103.02 ± 2.83	1.07	5	0.34
sIPSCs Dex – 100 nM	7.46 ± 0.60	7.95 ± 0.67	1.82	8	0.11	106.84 ± 3.33	2.05	8	0.07
mIPSCs	6.59 ± 0.50	6.92 ± 0.56	1.46	5	0.2	104.94 ± 3.30	1.5	5	0.19
sIPSCs Cort – 1 μM	10.59 ± 0.70	9.79 ± 0.61	1.53	7	0.17	93.40 ± 4.58	1.44	7	0.19

In 4 of 15 (∼27%) cells analyzed, Dex application caused an initial transient increase in sIPSC frequency (during the first 5 min of perfusion) into the submersion chamber (**Figure 5D**). It appeared that initial exposure to Dex caused bursting-like behavior in these cells; however, there were no significant differences in burst density (events per burst) or total number of bursts between control and Dex conditions (*t* = 0.5702, df = 14, *p* = 0.5776).

To determine if the effects of Dex were dose-dependent, we tested the effects of low concentrations (10 and 100 nM) on sIPSC frequency, amplitude, and decay time. Half-maximal effective concentrations (EC_50_) for Dex and Cort effects on synaptic signaling in the hippocampus and amygdala have been reported as 50–350 nM with 100 nM and 1 μM used as a common dose (Wiegert et al., 2006; Di et al., 2009). We tested the effects of Dex on sIPSC frequency, amplitude, and decay time at concentrations of 10 nM, 100 nM, and 1 μM. Dex failed to significantly alter sIPSC frequency at both 10 nM (*p* = 0.44) and 100 nM (*p* = 0.79), but significantly decreased sIPSC frequency at 1 μM (*p* = 0.01) (**Table 4**). Dex had no effect on sIPSC amplitude at 10 nM (*p* = 0.69), 100 nM (*p* = 0.22), or 1 μM (*p* = 0.57) (**Table 5**). Dex also had no effect on sIPSC decay time at 10 nM (*p* = 0.43), 100 nM (*p* = 0.11), or 1 μM (*p* = 0.07) (**Table 6**). Because Dex at 1 μM was the only concentration to produce a significant change in frequency of spontaneous inhibitory signaling, all experiments were performed at this concentration.

To determine the effect of Dex on mIPSCs, recordings were performed on MEC – LII principal cells in the presence of TTX, a voltage-gated sodium channel blocker (**Figure 5E**). Ten minutes of Dex application failed to significantly alter mIPSC frequency (*p* = 0.59; **Table 4**), amplitude (*p* = 0.65; **Table 5**), or decay time (*p* = 0.20; **Table 6**) (**Figures 5F,G**), indicating that Dex does not significantly alter presynaptic terminal-specific GABA release onto MEC – LII principal cells.

**FIGURE 6:**
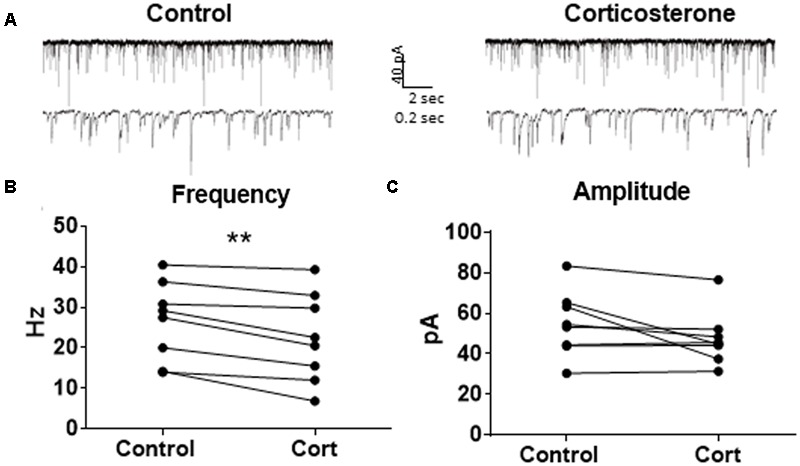
Corticosterone mimics the effect of dexamethasone on sIPSC frequency. **(A)** Twenty seconds (top) and 2 s (bottom) of sIPSC voltage-clamp recordings representative of control (left) and Cort (right) conditions. **(B)** Cort significantly decreased sIPSC frequency. **(C)** Cort had no effect on sIPSC amplitude (*n* = 8, ^∗∗^*p* < 0.01).

Because Dex is a synthetic glucocorticoid and GR-specific agonist, we tested Cort, the naturally circulating glucocorticoid in mice. Spontaneous IPSCs were recorded at a holding potential of -65 mV (**Figure 6A**). Ten minutes of Cort application significantly decreased sIPSC frequency (*p* = 0.01; **Table 4**), but did not significantly alter sIPSC amplitude (*p* = 0.09; **Table 5**) or decay time (*p* = 0.17; **Table 6**) (**Figures 6B,C**), indicating that Cort significantly decreased the frequency of spontaneous inhibitory signaling onto MEC – LII principal cells without significantly affecting amplitude or decay time of inhibitory synaptic events. Cort application mimicked the effect of Dex application on inhibitory signaling in MEC – LII.

### Pre-treatment With Dexamethasone Increased the Proportion of Cells With a Norepinephrine-Induced Increase in sIPSC Frequency

**FIGURE 7:**
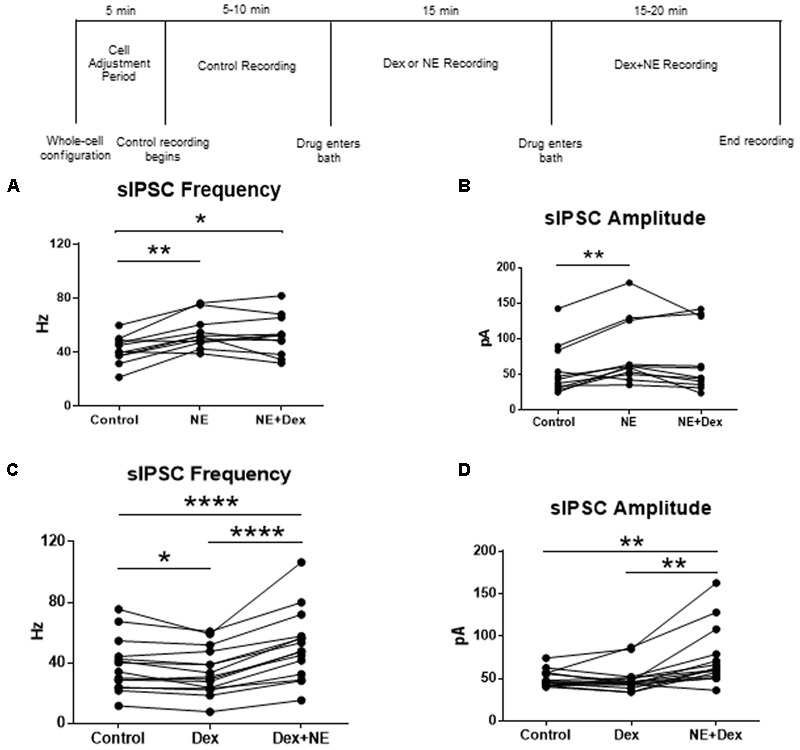
Norepinephrine application before Dex blocked the decrease in sIPSC frequency. Dex pretreatment increased the proportion of MEC- LII cells affected by NE. **(A)** NE alone and NE+Dex significantly increased sIPSC frequency from control, but frequency in NE+Dex was not significantly different from NE alone. **(B)** NE alone significantly increased sIPSC amplitude when compared to control. NE increased sIPSC frequency by more than 15% in 10 of 13 cells recorded. The averages in each drug condition were divided into three groups: all cells (*n* = 13), NE-sensitive cells (*n* = 10), and NE-insensitive cells (*n* = 3). **(C)** Dex significantly decreased sIPSC frequency. Dex+NE significantly increased sIPSC frequency from Dex-alone and control in all cells tested. **(D)** NE+Dex significantly increased sIPSC amplitude from Dex-alone and control (^∗^*P* < 0.05, ^∗∗^*p* < 0.01, ^∗∗∗∗^*p* < 0.0001).

To test for differential effects of stress hormones applied together compared to the independent effects already shown, co-application of NE and Dex was used to test changes to sIPSCs in MEC-LII principal cells. In this design, control baselines were achieved prior to adding NE (100 μM) alone, and then Dex (1 μM) was added together with NE. Frequency of sIPSCs in both the NE alone and NE+Dex condition was significantly increased compared to the control condition, but NE+Dex was not significantly different from NE alone (repeated measures 1-way ANOVA, *p* = 0.002, Tukey *post hoc* comparison: control vs. NE = ^∗∗^, control vs. NE+dex = ^∗^, NE vs. NE+Dex was not significantly different) (**Figure 7A**). NE significantly increased amplitude from control conditions (repeated measures 1-way ANOVA, *p* = 0.007, Tukey *post hoc* comparison: control vs. NE = ^∗∗^, control vs. NE+dex = NS, NE vs. NE+dex = NS) (**Figure 7B**), NE had no effect on decay time compared to the control condition (repeated measures 1-way ANOVA, *p* = 0.37). Consistent with our previous results, NE significantly increased sIPSC frequency in 10 of 13 cells recorded, and Dex did not affect sIPSC frequency in the 3 cells that were unresponsive to NE. These results suggest that NE blocked the decrease in sIPSC frequency caused by Dex.

To confirm that the co-administration of Dex and NE is consistent with independent application of each drug, the order of drug application was reversed. After recording control baselines, Dex (1 μM) was perfused for approximately 15 min prior to NE (100 μM)+Dex bath application. Frequency of sIPSCs was significantly different in all conditions tested (repeated measures 1-way ANOVA, *p* < 0.0001, Tukey *post hoc* comparison: control vs. Dex = ^∗^, control vs. NE+Dex = ^∗∗∗∗^, Dex vs. NE+Dex = ^∗∗∗∗^) (**Figure 7C** and **Table 7**). Amplitude of sIPSCs in the NE+Dex condition was significantly increased compared to both the control and Dex alone condition (repeated measures 1-way ANOVA, *p* = 0.001, Tukey *post hoc* comparison: control vs. exp = NS, control vs. NE+Dex = ^∗∗^, Dex vs. NE+Dex = ^∗∗^) (**Figure 7D** and **Table 8**). Decay time of sIPSCs in the NE+Dex condition was significantly increased compared to both the control and Dex-alone condition (repeated measures 1-way ANOVA, *p* < 0.0001, Tukey *post hoc* comparison: control vs. dex = NS, control vs. NE+Dex = ^∗∗∗^, Dex vs. NE+Dex = ^∗∗^). Surprisingly, NE increased sIPSC frequency from Dex alone conditions by greater than 15% in all (15 of 15) cells recorded (**Figure 7C**). In control vs. NE alone, 3 of 13 cells did not have a change in sIPSC frequency >15%, whereas NE induced a greater than 15% increase in all 15 cells when first primed with Dex for approximately 15 min. The increase in proportion of NE-affected cells from 10 of 13 to 15 of 15 is statistically significant (Chi-square expected vs. observed, *p* < 0.05; **Table 9**).

**Table 7:** Effect of adrenergic receptor activation on IPSC frequency with and without Dex pre-treatment.

	Control (Hz)	NE/PHE (Hz)	NE/PHE+Dex (Hz)	*F*	*n*	*p*	Con vs. NE/PHE	Con vs. NE/PHE+Dex	NE/PHE vs. NE/PHE+Dex
NE+Dex	42.39 ± 3.89	54.32 ± 3.59	52.31 ± 4.16	11.77	11	0.002^∗^	^∗∗^	^∗^	NS
PHE+Dex	29.93 ± 4.33	36.39 ± 5.70	n/a	*t* = 1.89	df = 9	0.09	NS	n/a	n/a

	**Control (Hz)**	**Dex (Hz)**	**Dex+NE/PHE (Hz)**	***F***	***n***	***p***	**Con vs. Dex**	**Con vs. Dex+NE/PHE**	**Dex vs. Dex+NE/PHE**

Dex+NE	37.83 ± 4.49	34.24 ± 3.94	51.25 ± 5.87	42.61	15	<0.0001^∗^	^∗^	^∗∗∗∗^	^∗∗∗∗^
Dex+PHE	25.62 ± 2.82	23.69 ± 2.63	37.79 ± 3.37	51.73	15	<0.0001^∗^	^∗∗^	^∗∗∗∗^	^∗∗∗∗^

*^∗^*p* < 0.05, ^∗∗^*p* < 0.01, ^∗∗∗∗^*p* < 0.0001.*

**Table 8:** Effect of adrenergic receptor activation on IPSC amplitude with and without Dex pre-treatment.

	Control (pA)	NE/Agonist (pA)	NE/Agonist+Dex (pA)	*F*	*n*	*p*	Con vs. NE/PHE	Con vs. NE/PHE+Dex	NE/PHE vs. NE/PHE+Dex
NE+Dex	57.90 ± 9.34	80.61 ± 11.57	68.58 ± 13.56	7.11	11	0.007^∗^	^∗∗^	NS	NS
PHE+Dex	57.09 ± 4.02	60.66 ± 9.19	n/a	*t* = 0.48	df = 9	0.64	NS	n/a	n/a

	**Control (pA)**	**Dex (pA)**	**Dex+NE/agonist (pA)**	***F***	***n***	***p***	**Con vs. Dex**	**Con vs. Dex+NE/PHE**	**Dex vs. Dex+NE/PHE**

Dex+NE	49.38 ± 2.52	49.60 ± 4.04	73.63 ± 8.72	13.44	15	0.001^∗^	NS	^∗∗^	^∗∗^
Dex+ PHE	52.75 ± 3.74	51.55 ± 4.19	59.62 ± 5.10	6.14	15	0.01^∗^	NS	^∗^	^∗^

*^∗^*p* < 0.05, ^∗∗^*p* < 0.01.*

**Table 9:** Chi-square comparison of NE-alone vs. Dex-primed NE.

Chi-square	Increased frequency	No change in frequency	*X*^2^	df	*P*-Value	Significance
NE-Alone	10	3	3.88	1	0.05–0.02	^∗^
Dex-Primed	15	0				

*^∗^*p* < 0.05.*

### Pre-treatment With Dexamethasone Increased the Proportion of Cells With a Phenylephrine-Induced Increase in sIPSC Frequency

**FIGURE 8:**
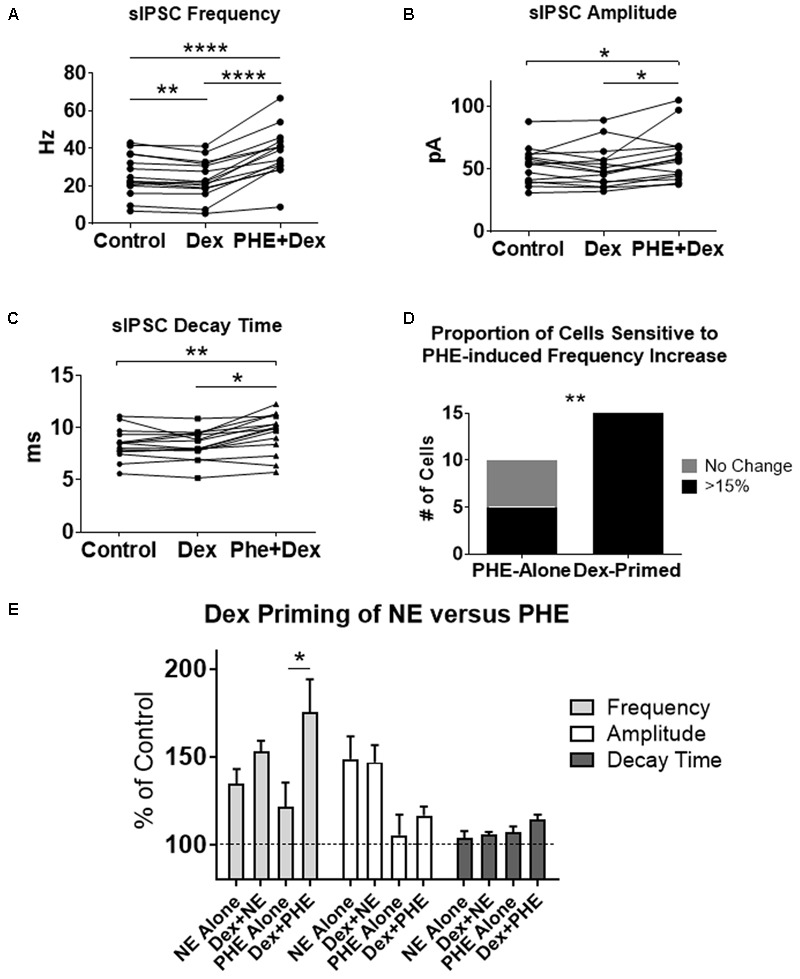
Dexamethasone pre-treatment increased the proportion of MEC-LII principal cells affected by phenylephrine. **(A)** Dex significantly decreased sIPSC frequency, and PHE+Dex significantly increased sIPSC frequency from Dex-alone and control (*n* = 15). **(B)** PHE+Dex significantly increased amplitude from Dex-alone and control. **(C)** PHE+Dex significantly increased decay time from Dex-alone and control. **(D)** Chi-square analysis of proportion of PHE-sensitive and insensitive cells in the PHE-alone condition vs. the Dex-priming condition. **(E)** Comparison of NE and PHE effect on sIPSC frequency, amplitude and decay time when applied alone and when cells were pre-treated with Dex. (^∗^*p* < 0.05, ^∗∗^*p* < 0.01, ^∗∗∗^*p* < 0.001, ^∗∗∗∗^*p* < 0.0001).

To test if the Dex-induced increase in cell proportion sensitive to an NE-induced increase in sIPSC frequency was α1-AR-mediated, slices were perfused with Dex (1 μM) for approximately 15 min prior to PHE (100 μM) entering the bath (**Figure 8**). Frequency of sIPSCs in all conditions was significantly different (repeated measures 1-way ANOVA, *p* < 0.0001, Tukey *post hoc* comparison: control vs. Dex = ^∗∗^, control vs. PHE+Dex = ^∗∗∗∗^, Dex vs. PHE+Dex = ^∗∗∗∗^) (**Figure 8A** and **Table 7**). Amplitude of sIPSCs in the PHE+Dex condition was significantly increased compared to the control condition (repeated measures 1-way ANOVA, *p* = 0.01, Tukey *post hoc* comparison: control vs. Dex = not significant, control vs. PHE+Dex = ^∗^, Dex vs. PHE+Dex = ^∗^) (**Figure 8B** and **Table 8**). Decay times of sIPSCs in the Dex+PHE condition were significantly higher than both the control and Dex-alone condition (repeated measures 1-way ANOVA, *p* < 0.0001, Tukey *post hoc* comparison: control vs. Dex = NS, control vs. PHE+Dex = ^∗∗^, Dex vs. PHE+Dex = ^∗∗∗^) (**Figure 8C**). Surprisingly, PHE increased sIPSC frequency from Dex-alone conditions by greater than 15% in all 15 of 15 cells recorded, (**Figure 8D**). In control vs. PHE-alone, 5 of 10 cells did not have a change in sIPSC frequency of >15%, whereas PHE induced a greater than 15% increase in all 15 cells if first primed with Dex for approximately 15 min. The increase in proportion of PHE-affected cells from 5 of 10 to 15 of 15 is statistically significant (Chi-square = 9.38, df = 1, *p* < 0.01) (**Figure 8D** and **Table 10**). Normalized sIPSC frequency changes in PHE primed with Dex vs. Dex-alone were significantly larger than PHE-alone vs. control (*p* = 0.04) (**Figure 8E**).

**Table 10:** Chi-square comparison of PHE-alone vs. Dex-primed PHE.

Chi-square	Increased frequency	No change in frequency	*X*^2^	df	*P*-Value	Significance
PHE-Alone	5	5	9.38	1	0.01–0.001	^∗∗^
Dex-Primed	15	0				

*^∗∗^*p* < 0.01.*

## Discussion

These findings demonstrate the ability of stress hormones to rapidly alter inhibitory signaling within MEC-LII circuits. The acute stress response includes activation of the HPA system to increase circulating glucocorticoids and the sympathetic nervous system to increase NE release into diffuse brain areas. NE application dramatically increased the frequency and amplitude of sIPSCs. Application of an α1-AR agonist, phenylephrine, was able to mimic the large increase in sIPSC frequency seen with NE in only half of the cells tested but failed to mimic the NE-induced increase in sIPSC amplitude; however, the α1-AR antagonist occluded the NE-induced increase in both frequency and amplitude of IPSCs. These results, together with the fact that α2 and β-AR activation caused little or no effect, suggest that the NE-induced increase in frequency and amplitude is primarily mediated by activation of α1-ARs. Application of NE also increased the frequency of mIPSCs indicating that GABA release from the presynaptic terminal increases even in the absence of cell spiking. In addition, NE significantly increased mEPSC decay time. The α1 or α2-AR agonist alone did not mimic the terminal-specific increase in mIPSC frequency, but the α2-AR agonist did significantly increase mIPSC decay time, mimicking the NE effect on decay time. This result suggest that α2-AR activation may specifically modulate post-synaptic GABA receptors. Because NE primarily affects frequency of both spontaneous and terminal-specific signaling seen in the MEC-LII principal cells, the frequency effect is likely localized to the pre-synaptic GABAergic cells located locally and sending inputs to the principal cells. Activation of the α1-ARs was necessary but not sufficient for the effect on amplitude of sIPSCs caused by NE application. This result suggest that the α1-ARs interact with another NE-activated pathway to likely increase post-synaptic GABA receptor currents. Interestingly, previous studies have indicated that acute stress, possibly through NE release, can mediate increases in GABA receptor expression (Cid et al., 2013). The effect of NE on GABA receptor expression in MEC remains to be determined.

Given the complex network of inhibitory inputs and the selective nature of the distinct interneuron classes in terms of targeting different principal cell classes, varying expression of ARs in the different interneuron classes could account for the NE-sensitive and NE-insensitive groups seen in these experiments. No matter the mechanism, NE causes a strikingly large increase in inhibitory signaling in MEC-LII. The reason for this level of increased inhibitory tone is unclear, though it does not necessarily follow that increased inhibition in MEC-LII decreases functional output. In fact, sufficiently large hyperpolarizing pulses in MEC-LII stellate cells cause rebound spiking through activation of HCN channels (*I*_h_) (Alonso and Klink, 1993; Hasselmo et al., 2007; Hasselmo and Shay, 2014; Shay et al., 2016), meaning inhibition can be readily converted to an increase in cellular output, though this remains to be tested.

### Cell-Type Specific NE Sensitivity

Several lines of evidence suggest that different cell types are differentially sensitive to NE. First, average baseline membrane potential in NE-sensitive cells was significantly hyperpolarized compared to NE-insensitive cells, suggesting cell type differences consistent with the idea that different cell types in MEC-LII have different resting membrane potentials (Fuchs et al., 2016). Second, average baseline input resistance of NE-insensitive cells was significantly greater compared to cells that show NE-induced changes in inhibitory signaling, and finally average sag was close to zero in all three cells that failed to show an NE-induced increase in sIPSC frequency, though the average was not significantly different from the NE-sensitive group. This difference in intrinsic properties could be an indication that the NE-insensitive cells are a different cell type. True stellate cells are consistently measured as having the lowest input resistance and largest sag amplitude, while the true pyramidal cells have the highest input resistance and smallest sag amplitude, and the intermediate stellate and pyramidal cells measure on a gradient between the true stellate and pyramidal cell groups (Alonso and Klink, 1993; Fuchs et al., 2016). In this case, the true pyramidal cell class is the most likely subset of cells that fail to show NE-induced changes to inhibitory inputs, however, previous studies showed that true pyramidal cells were more hyperpolarized compared to our results (Fuchs et al., 2016). This study used a low Cl- rather than a high Cl- solution, which may contribute to the differences in membrane potential.

Glucocorticoids, both corticosterone, which activates both MRs and GRs and Dex, a GR-specific agonist, consistently and rapidly decreased the frequency of spontaneous inhibitory signaling in MEC-LII principal cells. MRs have a higher affinity for glucocorticoids and are bound and activated at lower concentrations than GRs, which have a much lower affinity for glucocorticoids, and GRs are not activated until concentrations are greatly increased above basal levels (De Kloet et al., 1998; Timmermans et al., 2013). Because the same effect on sIPSC frequency is seen in the presence of both Cort and Dex, the glucocorticoids likely exert this frequency modulation by acting through GRs rather than MRs. Because glucocorticoids did not alter miniature IPSC frequency or amplitude in MEC-LII principal cells and failed to alter IPSC amplitude, we conclude that GR activation resulting in decreased sIPSC frequency is not due to pre-synaptic GR-induced modulation of terminal-specific GABA release or post-synaptic GABA_A_ receptor modulation. It is more likely that activation of membrane GR occurs at the pre-synaptic cell to decrease frequency of spike-evoked GABA release. However, we cannot rule-out the idea that post-synaptic GR activation leads to retrograde release of endocannabinoids or nitric oxide (Makara et al., 2007). Depolarization-induced suppression of inhibition (DSI) is a commonly known mechanism in which endocannabinoid release from the post-synaptic cell acts on pre-synaptic endocannabinoid receptors to decrease GABA release. Glucocorticoids are also known to trigger release of NO through activation of GR, which acts on pre-synaptic GABAergic cells to increase spiking (Nahar et al., 2015).

We show that co-administration of the two stress hormones, Dex and NE, caused differential responses depending on the order of application. NE application prior to Dex application essentially blocked the decrease in sIPSC frequency effect of Dex. However, when Dex was perfused for 15 min prior to NE, Dex enhanced the NE effect and every cell responded to NE with a larger than 15% increase in sIPSC frequency, and this Dex priming effect was mimicked by the α1-AR agonist, phenylephrine. These findings are novel in three ways. First, they show that NE application can block the glucocorticoid effect, which suggests the effects may utilize similar pathways. Second, they show that a subset of cells in MEC-LII are unresponsive to NE without prior activation of GR. Third, incubation of MEC-LII principal cells in Dex eliminates this set of non-responders so that all principal cells in MEC-LII show an NE-induced increase in sIPSC frequency. Thus, activation of GR by Dex rapidly primes a subset of cells to become responsive to NE application that were previously unaffected. Previous tests on a mix of cells from MEC-LII and LIII in rats less than 3 weeks of age when MEC circuitry is not yet fully developed, showed that 100% of the principal cells were affected by NE (Lei et al., 2007). The present study is the first test of NE’s effects when recording exclusively from cells in dorsal MEC-LII in animals old enough to have fully matured grid cells (Langston et al., 2010). Furthermore, the known differences between LII and LIII including cell type, projection routes, intrinsic characteristics, and inhibitory networks, necessitated testing the effect of NE on solely MEC-LII principal cells in mature animals.

Though we are yet to investigate the mechanism of this priming effect, previous evidence suggests that effects of glucocorticoids can be mediated through activation of NE receptors. For example, in the amygdala, the emotional memory enhancing effects of glucocorticoids require NE interaction in the basolateral amygdala and this effect is mediated by β-AR activation (Quirarte et al., 1997; Roozendaal et al., 2002), but also includes an interaction with α1-ARs (Ferry et al., 1999a,b; Roozendaal et al., 2002). Furthermore, in the hypothalamus, GR activation by Dex internalizes α1-ARs to make the cells unresponsive to an NE-induced increase in sIPSC frequency (Tasker, SFN abstract, 2015). Mechanistically, this suggests that Dex application can interact with ARs to influence their sub-cellular positioning. Our results would require activation of GR to cause AR membrane insertion in cells with mostly internalized α1-ARs. Evidence suggests that sub-cellular positioning can determine effectiveness and affinity of AR agonists. In Cos-7 cell cultures, α1_A_-ARs are primarily located internally while α1_B_-ARs are primarily located in the cell membrane (Tsujimoto et al., 1998). Furthermore, α1-AR affinity for an agonist depends on receptor insertion in the membrane (Sugawara et al., 2002). If GR activation causes insertion of ARs into the membrane in the subset of cells that were previously unresponsive to NE, it could account for the priming effect observed. Thus, the mechanisms of interactions between GRs and ARs requires further investigation.

Our results demonstrate that approximately one quarter of MEC-LII principal cells are insensitive to NE/PHE-induced frequency increases. We hypothesize that this subset of NE/PHE-insensitive cells are true pyramidal cells. 5HT_3A_ interneurons exclusively inhibit true pyramidal cells in MEC-LII, and PV^+^ fast-spiking interneurons synapse onto all MEC-LII principal cells except true pyramidal cells (Fuchs et al., 2016). Thus, sensitization of true pyramidal cells could be explained by GR-induced α1-AR membrane insertion in pre-synaptic 5HT_3A_ interneurons that do not express membrane-bound α1-ARs without GR-activation.

Whatever the mechanism, we show that glucocorticoids, at levels normally seen in the circulating blood of an organism recently exposed to a stressor (Di et al., 2003, 2005), rapidly activate GRs and interact with NE to affect inhibitory signaling within MEC-LII. Rapid effects of membrane GRs are seen in multiple species and are considered an evolutionarily conserved mechanism (Dallman, 2005), indicating that this response is adaptive and may be an effective means of dealing with stressful stimuli. Since the stress response is evolutionarily conserved, it is likely that the subset of NE-insensitive cells serves as an advantage to the organism in normal circumstances, though it remains to be determined if these cells are sensitized to respond to NE only in stressful situations, and what effect this has on the organism’s behavior. Previous studies have indicated a gradient of inhibition in the dorsal-ventral MEC axis, and this gradient of inhibition correlates to an increase in spacing of grid cell firing (Beed et al., 2013).

Therefore, these demonstrated effects on inhibitory inputs may affect spacing of grid cell firing. Furthermore, given the known importance of inhibitory inputs to oscillatory activity in MEC-LII, a change in signal-to-noise ratio of inhibitory inputs could dramatically alter theta-nested gamma known to be crucial for spatial memory processing (Chrobak and Buzsaki, 1998; Quilichini et al., 2010; Colgin, 2015, 2016), and suggest a possible mechanism for stress modulation of spatial memory formation. In addition, the effects on inhibitory signaling demonstrated here are also likely important to pathological functions. Interestingly, low magnesium-induced epileptic activity in the entorhinal cortex can be blocked by α1-AR activation following NE application (Stanton et al., 1987), suggesting that the observed increase in inhibition is important to suppress hyperactivity leading to epilepsy within known spatial processing circuits. Furthermore, noradrenergic innervation in the MEC is reduced in rodent models of AD (Chalermpalanupap et al., 2017; Rorabaugh et al., 2017). In addition, AD patients exhibit elevated cortisol levels (Masugi et al., 1989; Swaab et al., 1994; Lehallier et al., 2016), thus the balance of CORT/NE regulation of inhibitory circuitry is likely disrupted and may contribute to AD pathologies and deficits in spatial memory. Ultimately, a better understanding of the connection between stress and spatial memory processing has implications for both our understanding and ability to treat populations affected by epilepsy, post-traumatic stress disorder, Alzheimer’s disease, and learning and memory disorders.

## Author Contributions

JPH completed the experimental design and performance, data analysis, and manuscript preparation. LAS contributed to the experimental design and manuscript preparation.

## Conflict of Interest Statement

The authors declare that the research was conducted in the absence of any commercial or financial relationships that could be construed as a potential conflict of interest.
